# CONTEXTUAL FACTORS THAT SHAPE EXPERIENCES OF SEXUAL INTIMACY AMONG LATER-LIFE COUPLES

**DOI:** 10.1093/geroni/igad104.1711

**Published:** 2023-12-21

**Authors:** Jeremy Yorgason, Stephanie Wilson, Hui (Cathy) Liu

## Abstract

Sexuality is an important aspect of romantic dyadic relationships. Although people experience physical changes with age that result in changes in sexual interactions, the sexual aspect of couple relationships remain important. Research examining sexuality among later life couples is maturing, allowing investigators to explore more nuanced questions about couple contexts that shape sexual experiences. The current group of papers address some of these important contextual factors that can impact the couple sexual relationship, including sexual motives among same and different sex couples, personality characteristics and how they relate to sexual interactions, how couples’ sexual interactions are affected by transitions such as menopause, and how sleep patterns among caregivers are related to sexual interactions. Presenters in this session draw upon national studies of older adults including NSHAP, MIDUS, and HARP. They used advanced statistical modeling to explore their questions. Findings suggest that self-determined sexual motives among same and different sex couples were associated with better marital quality and lower depression. The menopause transition, along with relationship transitions in later life, were associated with sexual interactions. Conscientiousness and neuroticism were associated with sexual pleasure and emotional satisfaction, along with preferences regarding physical touch within the relationship. Among caregivers, sleep duration was less important; sharing a bed with a care-recipient partner was associated with physical pleasure; and napping was negatively associated with sexual quality. In summary, various factors impact the sexual interactions of older couples in different ways. Findings from these papers given nuanced insight into a number of important contextual factors.

